# Supplementary figures and images for: Soluble MAC is primarily released from MAC-resistant bacteria that potently convert complement component C5
Source: eLife. 2022 Aug 10;11:e77503. doi: 10.7554/eLife.77503 (PMC9402229; doi:10.7554/eLife.77503)

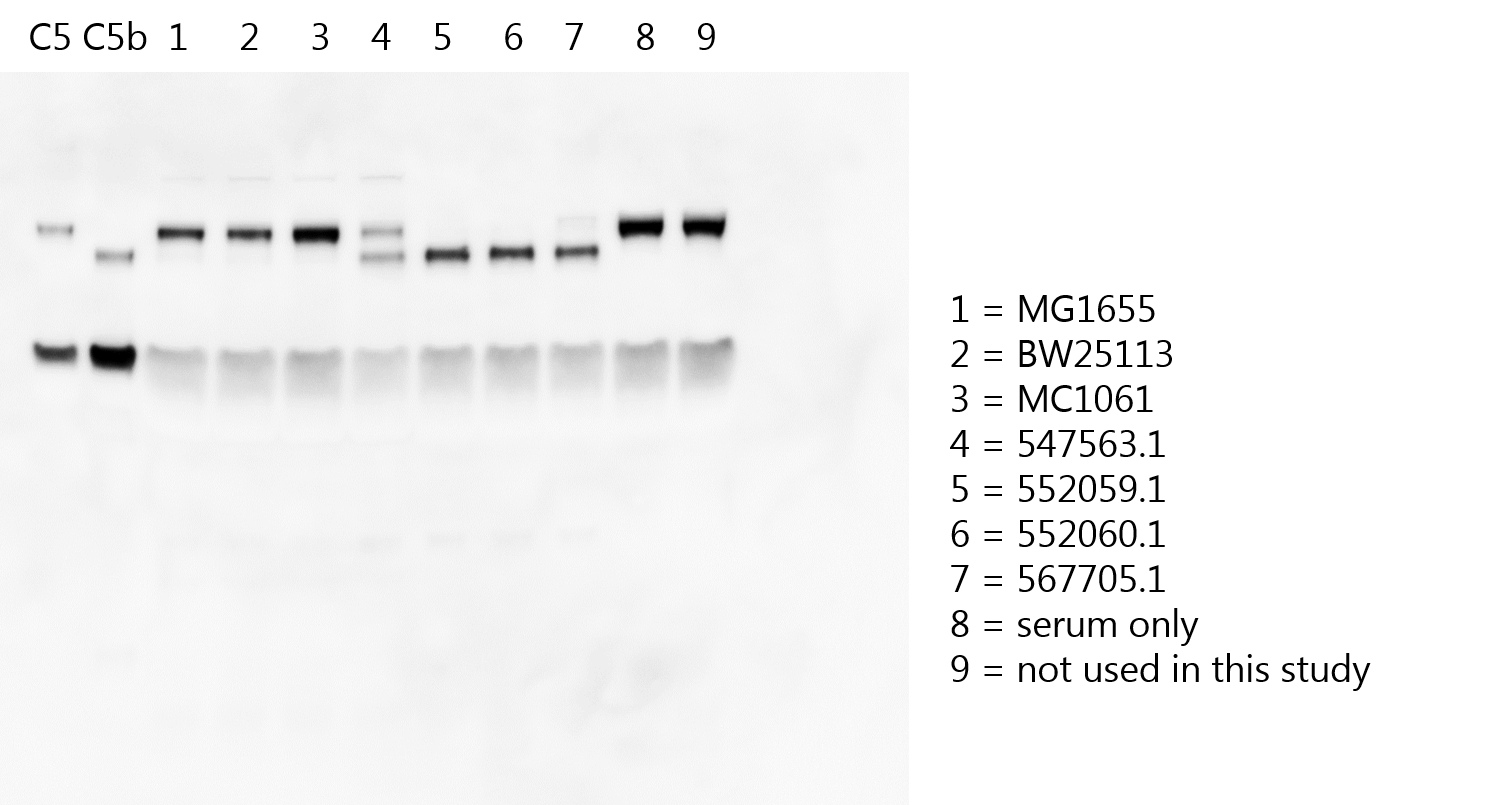

Supplement: Figure 3—source data 1. — The supernatant was analyzed by Western blotting for C5. Serum without bacteria (serum only) was taken as control for the absence of C5 conversion. Ten nM C5 or pC5b6 were loaded as positive controls for C5 and C5b. [file elife-77503-fig3-data1.jpg]

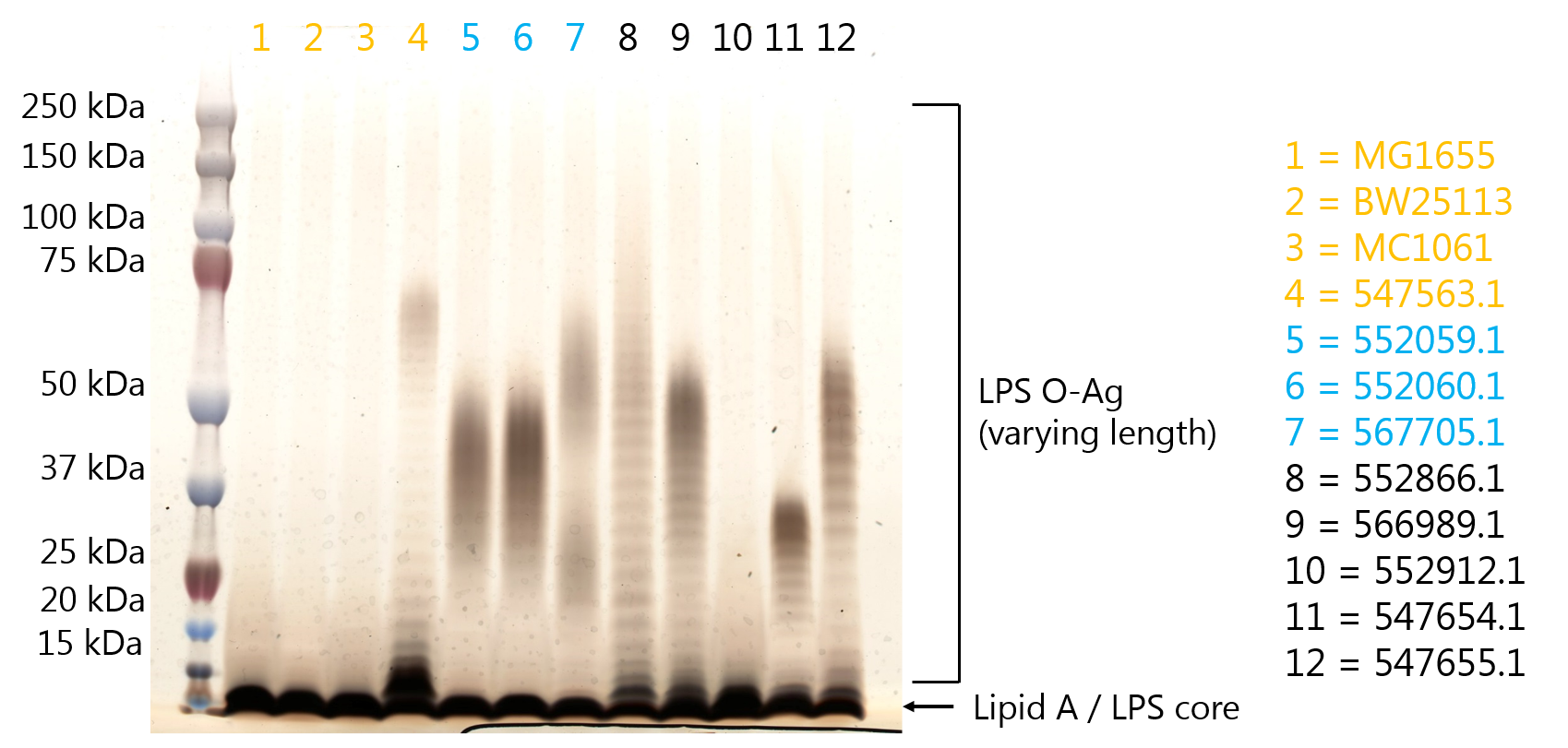

Supplement: Figure 3—figure supplement 1—source data 1. — Orange strains are MAC-sensitive (MAC-sens), blue strains MAC-resistant (MAC-res), and black strains complement-resistant (comp-res). A dual-color marker was loaded as control (on the left). The silver stain was previously shown in part (only for silver MAC-resistant and MAC-sensitive E. coli strains) in Doorduijn et al., 2021. [file elife-77503-fig3-figsupp1-data1.jpg]

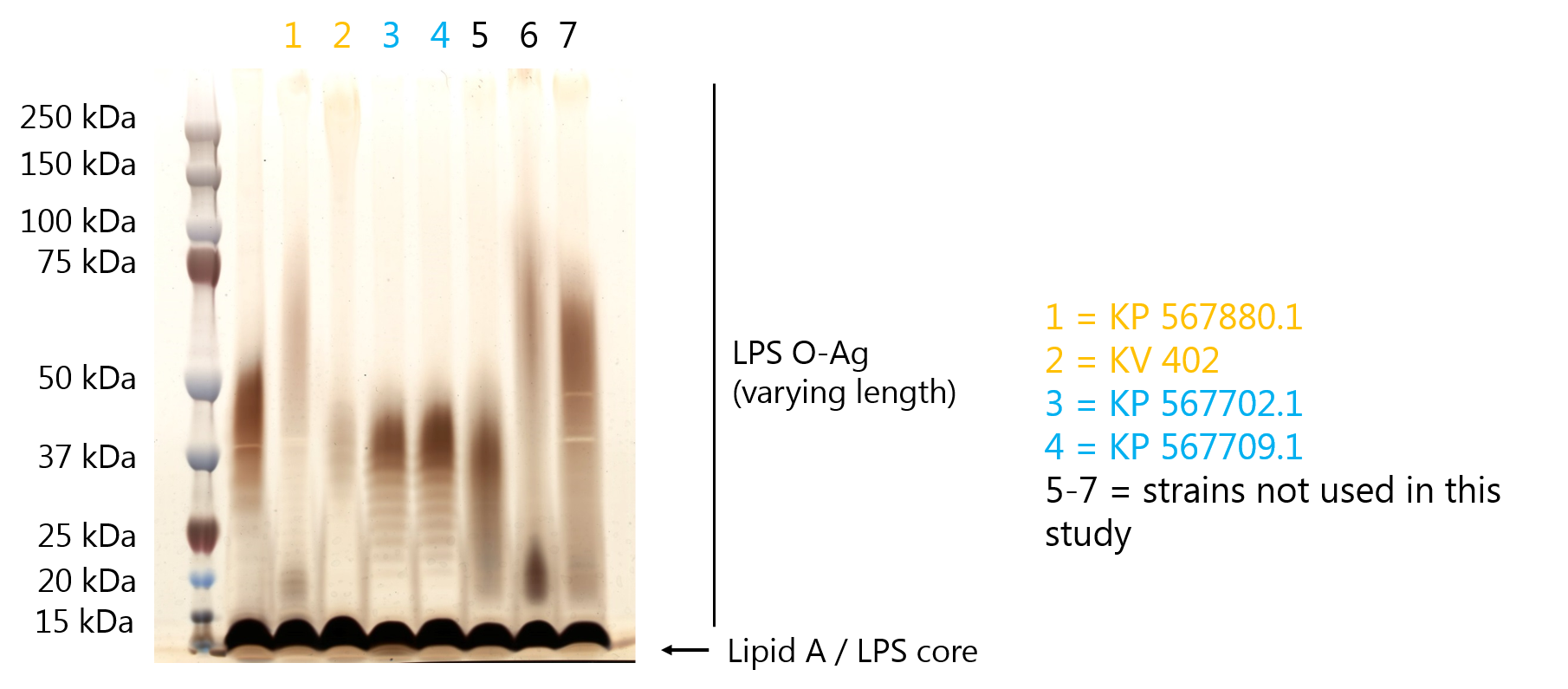

Supplement: Figure 3—figure supplement 1—source data 2. — Orange strains are MAC-sensitive (MAC-sens), blue strains MAC-resistant (MAC-res), and black strains complement-resistant (comp-res). A dual-color marker was loaded as control (on the left). [file elife-77503-fig3-figsupp1-data2.jpg]

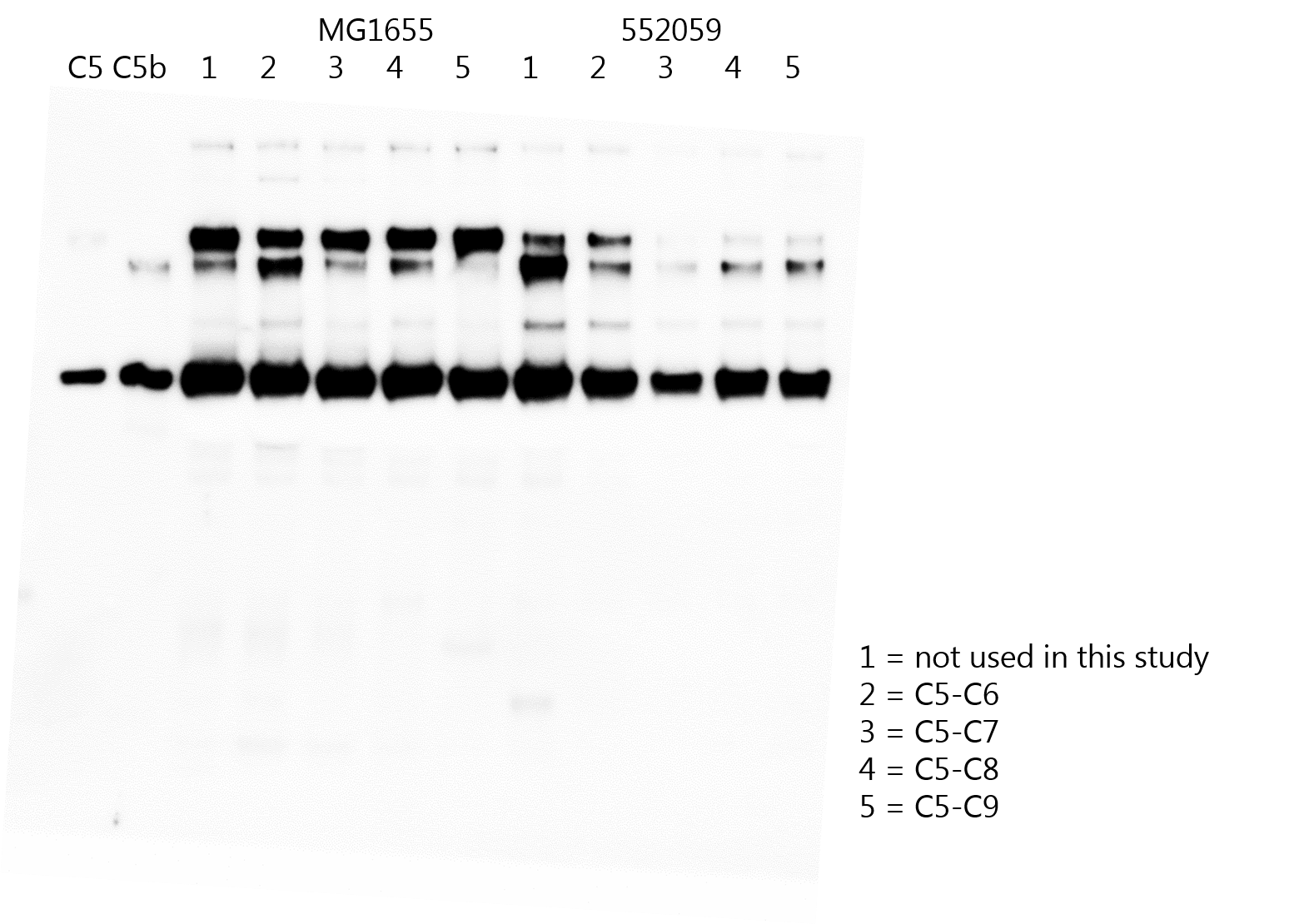

Supplement: Figure 4—source data 1. — Next, bacteria were washed and 5×10^8^ bacteria/ml were incubated with alternative pathway (AP) convertase components (5 µg/ml FB and 0.5 µg/ml FD) and 100 nM C5 and C6 (2); 100 nM C5, C6, and C7 (3); 100 nM C5, C6, C7, and C8 (4) or 100 nM C5, C6, C7, C8, and 1000 nM C9 (5). The supernatant was collected after 60 min by centrifugation and analyzed by Western blotting for C5. Ten nM C5 or pC5b6 were loaded as positive controls for C5 and C5b. [file elife-77503-fig4-data1.jpg]

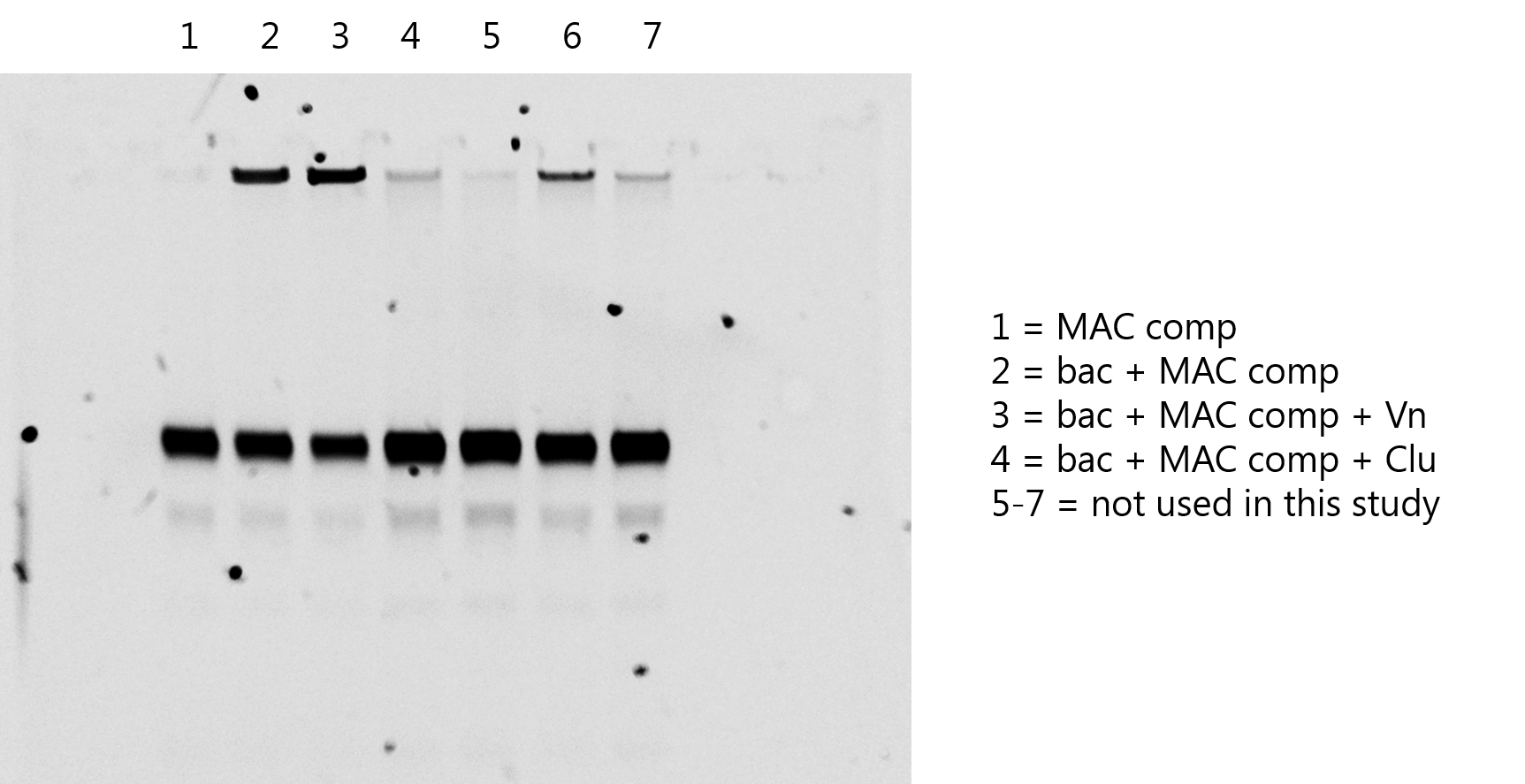

Supplement: Figure 5—figure supplement 2—source data 1. — Next, convertase-labelled bacteria (3.3×10^8^ per ml) were incubated with: alternative pathway (AP) convertase components (50 nM FB and 20 nM FD), MAC components C5-C9 (30 nM C5, 30 nM C6, 30 nM C7, 30 nM C8, and 300 nM Cy5-labelled C9, named MAC comp) with or without 133 nM vitronectin (Vn) or 133 nM clusterin (Clu). SDS-PAGE was performed to distinguish monomeric-C9 from polymeric-C9 by in-gel Cy5 fluorescence. [file elife-77503-fig5-figsupp2-data1.jpg]

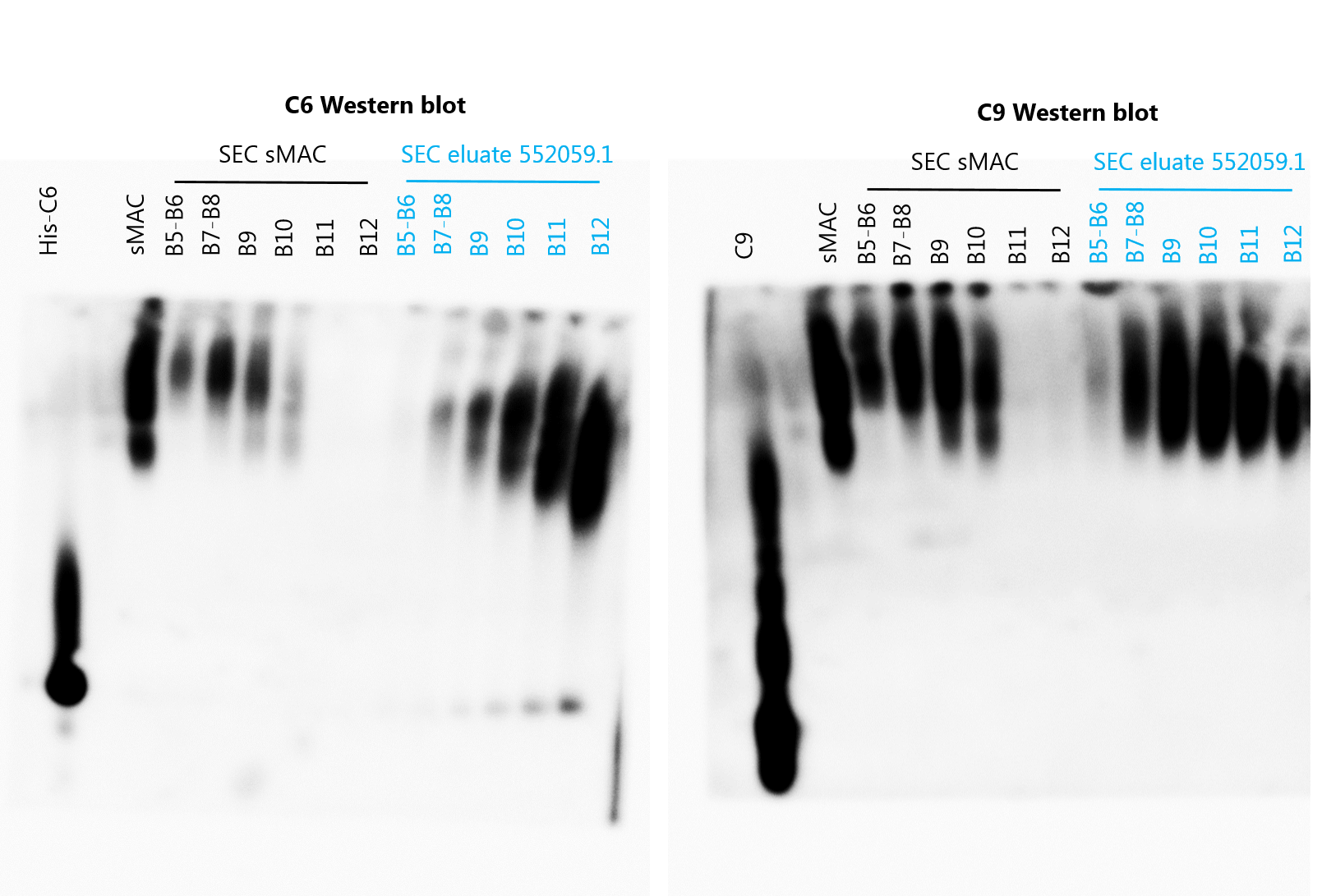

Supplement: Figure 6—source data 1. — sMAC in the supernatant was captured with HisTrap beads and eluted. Concentrated eluate was fractionated by size exclusion chromatography (SEC) on a Superose 6 column. Fifty µg commercially available sMAC (Complement Technology) was also analyzed as control. Blue-native PAGE (BN-PAGE) was performed with pooled (B5+B6 and B7+B8) or individual (B9-B12) SEC fractions and analyzed by Western blotting for C6 (left) and C9 (right). Black fractions represent SEC fractions commercially available sMAC, blue fractions represent SEC fractions of serum incubated with MAC-res E. coli. Two µg of purified sMAC and His-C6 or His-C9 were loaded as control. [file elife-77503-fig6-data1.jpg]
